# Superelasticity of Plasma‐ and Synthetic Membranes Resulting from Coupling of Membrane Asymmetry, Curvature, and Lipid Sorting

**DOI:** 10.1002/advs.202102109

**Published:** 2021-09-26

**Authors:** Jan Steinkühler, Piermarco Fonda, Tripta Bhatia, Ziliang Zhao, Fernanda S. C. Leomil, Reinhard Lipowsky, Rumiana Dimova

**Affiliations:** ^1^ Theory and Bio‐Systems Max Planck Institute of Colloids and Interfaces Science Park Golm Potsdam 14424 Germany; ^2^ Department of Physical Sciences Indian Institute of Science Education and Research Mohali Sector 81, Knowledge City, Manauli SAS Nagar Punjab 140306 India; ^3^ Departamento de Biofísica Universidade Federal de São Paulo São Paulo 043039‐032 Brazil; ^4^ Present address: Department of Biomedical Engineering Northwestern University Evanston IL 60657 USA; ^5^ Present address: Leibniz Institute of Photonic Technology Jena 07745 Germany

**Keywords:** giant plasma membrane vesicles, lipid domains, micropipette, plasma membrane, spontaneous curvature, superelasticity, synthetic biology

## Abstract

Biological cells are contained by a fluid lipid bilayer (plasma membrane, PM) that allows for large deformations, often exceeding 50% of the apparent initial PM area. Isolated lipids self‐organize into membranes, but are prone to rupture at small (<2–4%) area strains, which limits progress for synthetic reconstitution of cellular features. Here, it is shown that by preserving PM structure and composition during isolation from cells, vesicles with cell‐like elasticity can be obtained. It is found that these plasma membrane vesicles store significant area in the form of nanotubes in their lumen. These act as lipid reservoirs and are recruited by mechanical tension applied to the outer vesicle membrane. Both in experiment and theory, it is shown that a “superelastic” response emerges from the interplay of lipid domains and membrane curvature. This finding allows for bottom‐up engineering of synthetic biomaterials that appear one magnitude softer and with threefold larger deformability than conventional lipid vesicles. These results open a path toward designing superelastic synthetic cells possessing the inherent mechanics of biological cells.

## Graphical abstract

During their lifecycle, cells undergo remarkable deformations. The protective membrane holding together the cell (plasma membrane) must allow for such deformations. Here, plasma membrane extracts that retain some of the cellular deformability are studied. The underlying physics of the elastic response is identified and a synthetic biomaterial one magnitude softer and threefold more deformable than conventional synthetic cells is engineered.

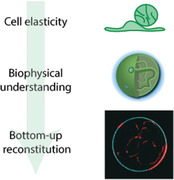

## 1 Introduction

The concept of compartmentalization is central to living systems. A ubiquitous compartment‐forming element in cells are lipid bilayers, with the most prominent example of the outer cell membrane or plasma membrane (PM). The PM is a complex biomaterial that has to support dynamic shape changes of the cell and its mechanical failure would result in cell death, highlighting the importance of PM mechanical properties. PM elasticity has been probed using micropipettes,^[^ ^1^ ^]^ tube‐pulling, and atomic force microscopy indentation experiments.^[^ ^2^, ^3^ ^]^ These methods probe the in situ response of the PM to mechanical challenges and are thus informative of the physiologically relevant response in the context of the living cell. For example, in situ experiments probe the PM adhesion to the cell cortex and recruitment of membrane material via exocytosis.^[^ ^4^ ^]^ This interwinding represents a challenge for reductionist approaches that try to rebuild some of the cellular functionality for “synthetic cells,” because engineering approaches rely on the definition of well‐characterized parts. Here, we address these challenges by isolation of plasma membrane sheets by chemical induction, a method that has been used before to study phase behavior^[^ ^5^ ^]^ and bending rigidity of plasma membranes.^[^ ^6^ ^]^ In contrast to previous studies, we describe conditions that lead to the reconstitution of large‐scale area reservoirs. Tension‐induced recruitment of these area reservoirs proceeds by an apparent, or superelastic, response far exceeding the deformability of usual lipid bilayers but representing a characteristic feature of living cell plasma membranes. By detailed comparison of experiment and theory, we isolate the curvature‐elastic parameters that allow for the observed superelastic behavior. Informed by these results we engineered a lipid‐only vesicle analogue that appears one magnitude softer and with threefold larger deformability than conventional lipid vesicles, thus bringing synthetic biology approaches one step forward toward building synthetic cells.

## 2 Results

### 2.1 Isolation of Structurally Conserved Plasma Membrane Leads to Formation of Vesicles with Cell‐Like Elastic Response

**Figure 1:**
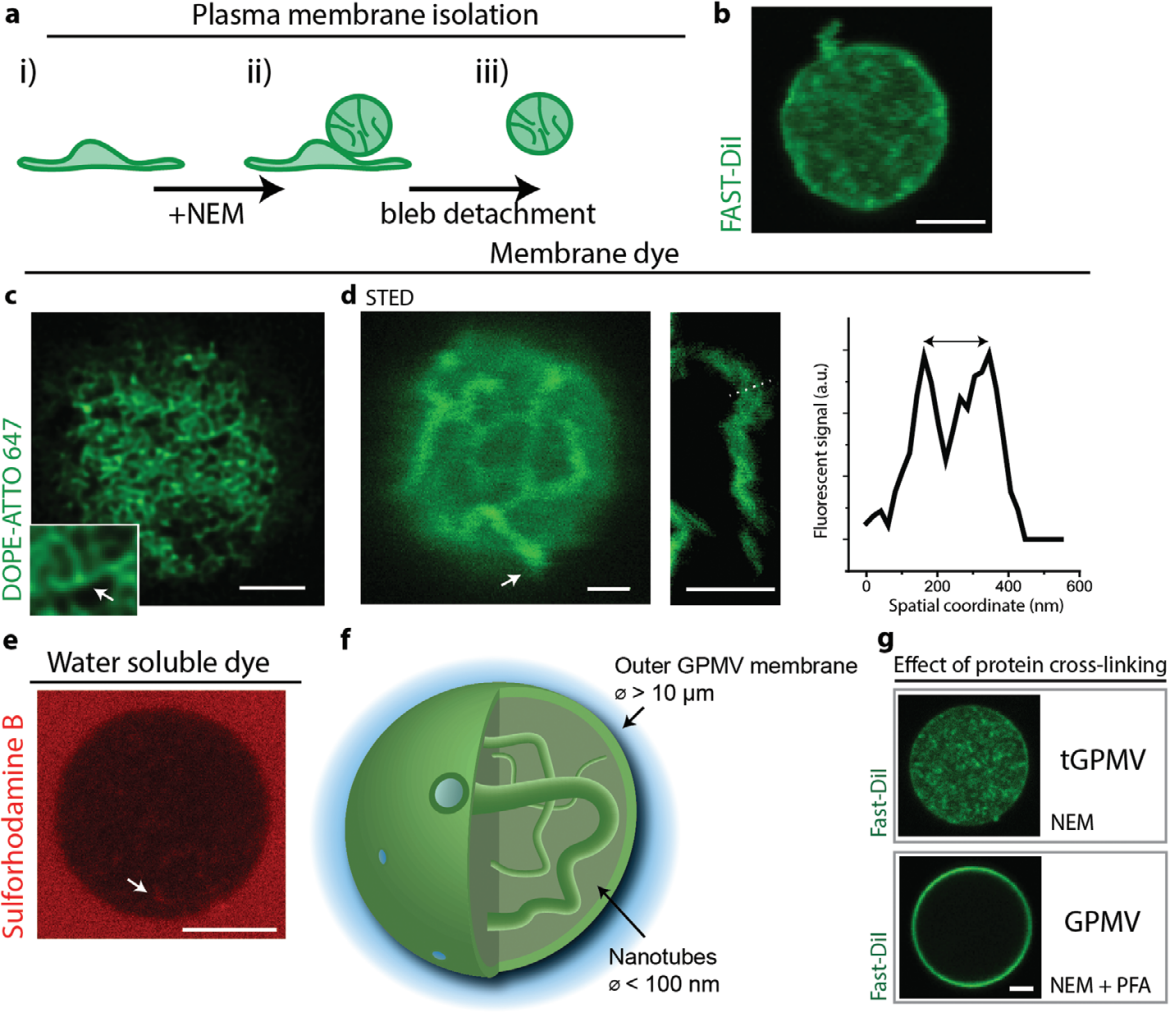
a) Extraction and isolation of plasma membrane by incubation of U2OS cells with *N*‐ethylmaleimide (NEM). Scale bar: 5 µm. b) Membrane extracts were isolated and labeled with fluorescent membrane dye (FAST‐DiI) (shown in green). c) High‐resolution imaging of the internal structures by deconvolution of confocal images reveals a dense lipid network. Possible three‐way branches are shown in the inset. d) STED microscopy of internal structures found in the lumen of the vesicles. Left: Nanotubes appear to be connected to the outer membrane. Middle: Image of one tube. Right: Fluorescence intensity plot along the dotted line indicated on the middle image. The two peaks correspond to the nanotube walls and exemplify a nanotube radius of about 100 nm. Scale bars: 1 µm. e) Diffusion of water‐soluble dye added to the outer solution into the nanotubes. The arrow shows signal detected in the water‐filled nanotube interior. Scale bar: 5 µm. f) Sketch of the overall GPMV structure (vesicle and nanotube diameter not to scale). g) Effect of crosslinking chemical PFA on plasma membrane vesicles. We term tGPMV extracts to those that reconstitute a tubular lipid network. Scale bar: 5 µm.

After chemical cleavage of the cytoskeleton‐PM anchors induced by incubation with *N*‐ethylmaleimide (NEM), turgor pressure leads to the formation of spherical blebs called giant plasma membrane vesicles (GPMVs). These vesicles reconstitute PM lipids and membrane proteins,^[^ ^7^ ^]^ and encapsulate soluble cytosolic components (**Figure** **1**a and the Experimental Section).^[^ ^8^, ^9^ ^]^ GPMVs were isolated from the cells and stained with the membrane dye FAST‐DiI (see the Experimental Section for details). Unexpectedly, fluorescence intensity not only was observed on the outer (spherical) GPMV membrane segment, but also appeared in the vesicle lumen (Figure 1b). We observed this effect both when fluorescent dye was introduced to the cells before GPMV extraction or to unlabeled GPMVs after extraction. Deconvolution and super‐resolution imaging (stimulated emission depletion, STED) revealed the presence of a tubular lipid network which is contained within the isolated GPMVs and connected to the outer membrane (Figure 1c). In some vesicles, the tubular network was found to be very dense and possibly branched (Figure 1c inset). The diameter of the lipid nanotubes appeared to be mostly below the resolution limit of our STED setup (about 50 nm), but some tubes were wide enough to be directly resolved by STED measurements (Figure 1d). To aid interpretation of the imaging results, a water‐soluble dye was added to the outer aqueous solution. Fluorescence signal from the water channels formed by the nanotubes provided direct evidence for the tubular structure and connection to the outer membrane (sulforhodamine B dye in Figure 1e). Together, this confirms that GPMVs isolated in the conditions employed here, reconstitute a nanotubular lipid network (Figure 1f), which is similar to tubular structures found in cells.^[^ ^10^, ^11^ ^]^ Curvature elasticity arguments suggest that coexistence of the outer, weakly curved membrane segment, and the highly curved nanotubes requires that the membrane exhibits preferred, or spontaneous curvature, in the order of *m*_tubes_> 1/200 nm^−1^ = 5 µm^−1^, stabilizing the highly bent lipid bilayer.^[^ ^12^ ^]^ Similar spontaneous curvature values were shown to be generated by adsorption of curvature‐inducing BAR domain proteins to the bilayer.^[^ ^13^ ^]^ Indeed proteomics indicates that BAR‐domain proteins are present in GPMVs^[^ ^9^ ^]^ and are known drivers of membrane tubulation in vitro and in vivo.^[^ ^14^ ^]^

Consistent with protein coverage on the cytosolic leaflet of the nanotubes, addition of the protein crosslinker paraformaldehyde (PFA) led to crosslinking of the nanotubes. This effect was exploited to immobilize and thus obtain the high‐resolution images of the otherwise dynamically fluctuating nanotube network shown in Figure 1c,d (also see the Experimental Section).

However, if the crosslinking chemicals were present during plasma membrane extraction, the GPMVs interior appeared free from any fluorescence signal of membrane dye (Figure 1g and the Experimental Section), suggesting that the excess lipid material was retained inside the cells by the crosslinking agent. Consistent with previous reports,^[^ ^15^ ^]^ the vesicles isolated in crosslinking conditions sometimes appear to be permeable to the water‐soluble dye. However, this effect leads to homogenous dye distribution in the vesicle lumen and can be clearly distinguished from the structured signal from internal nanotubes shown in Figure 1e. Previous experiments using GPMVs were often conducted in the presence of crosslinkers,^[^ ^16^ ^]^ and to distinguish the protocol used here, we term vesicles reconstituting a tubular lipid network tGPMVs.

### 2.2 Tension Retracts Nanotube Network to Outer Membrane

**Figure 2:**
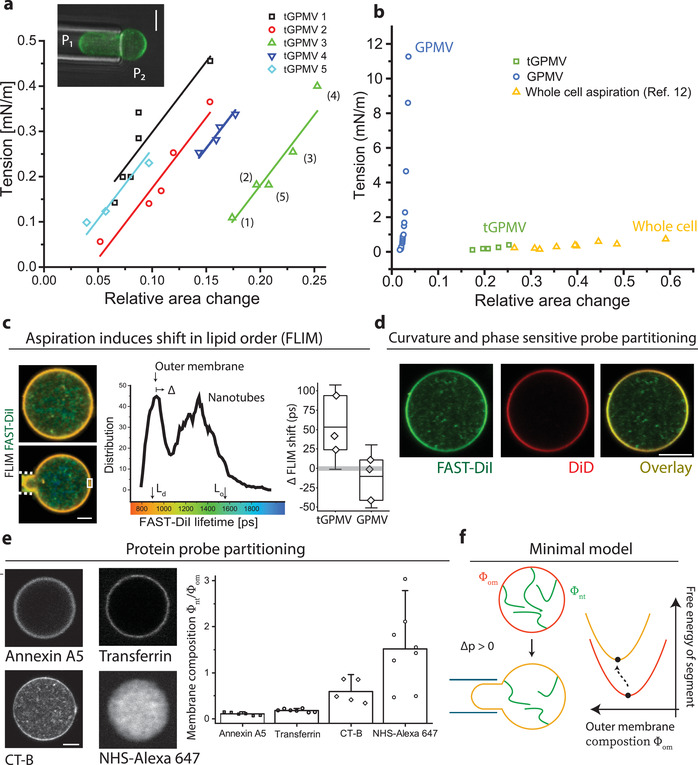
a) Vesicle projected (outer) area from micropipette aspiration of individual tGPMVs (color‐coded) at varying pressure differences Δ*P*  = *P*_2_  − *P*_1_, the tension is calculated from the Laplace pressure as $\frac{R_{p}\Delta P}{2(1-R_{p}/R_{v})}$, where *R*_p_ and *R*_v_ are the corresponding pipette and spherical membrane segment radii. The numbers in brackets indicate the experimental sequence of applied pressures. b) Comparison between apparent elastic moduli obtained for GPMVs and tGPMVs (measured here), and neutrophils (data from ref. [20]). c) FLIM data: color code indicates FAST‐DiI fluorescence lifetime in a free tGPMV (top image) and when aspirated (bottom image), white dashed lines indicate micropipette. The histogram shows lifetime distribution. Upon application of a low membrane tension (0.7 ± 0.3 mN m^−1^), the lifetime of the outer membrane segment (data collected in the region of interest marked by the white rectangle) is shifted to higher values by *Δ*. In the right panel, we compare this tension‐induced change (*Δ*) in tGPMVs and GPMVs. Each data point represents an independent experiment. Note that the fluorescence lifetime in the nanotube network (as evaluated from measurements in the central vesicle part) is not changing substantially (see Figure S2 in the Supporting Information). d) Co‐staining of the same tGPMV with membrane dyes FAST‐DiI and DiD (green and red channel) and overlay: DiD is expelled and sorted out from the nanotubes. e) Staining of the external leaflet of tGPMV with protein (primary amines) binding NHS‐Alexa 647 and fluorescently conjugated annexin A5, transferrin, and cholera toxin subunit B (CT‐B) (see the Experimental Section for details). Bar plot shows fluorescent signal ratio between outer membrane and the tubular network of tGPMVs. All vesicle diameters were between 10 and 30 µm. f) Sketch of the model of outer membrane segment with composition Φ_om_ and lipid nanotube reservoir of composition Φ_nt_. Aspiration leads to unfavorable lipid–lipid interaction in the outer membrane segment that contribute to the apparent elastic response.

As long as all membranes remain fluid and the nanotubes in the vesicle lumen are connected to the outer membrane, it should be possible to retract them to the outer vesicle membrane by exposing them to mechanical tension as previously shown.^[^ ^17^ ^]^ Indeed, when tGPMVs were aspirated into glass capillaries, similar to those used for elastic probing of cells^[^ ^18^ ^]^ and lipid vesicles,^[^ ^19^ ^]^ we were able to induce large‐scale elastic deformations corresponding to about 20–25% increase of the initial apparent outer membrane area (**Figure** **2**a). At a fixed tension tGPMVs remained stable with a constant membrane area of the outer segment and without indication of hysteresis along the trajectory (Figure 2a). For comparison, GPMVs that were isolated in conditions that do not reconstitute a nanotube network, appeared three orders of magnitude stiffer and did rupture at small area strains of about 3%, in a manner reminiscent of pure lipid bilayers^[^ ^19^ ^]^ (Figure 2b). When compared to literature data of micropipette aspiration of neutrophil cells,^[^ ^18^ ^]^ it becomes clear that tGPMV elasticity appears to be much more similar to that of intact cells rather than other biomimetic systems. While elasticity and area regulation in cells is certainly orchestrated by a range of (active) processes, including the actomyosin cortex and exocytosis of small vesicles, our results suggest that the plasma membrane composition and morphology itself is poised to exhibit an elastic response matching the cortical tension. While this behavior has interesting implications for cell biology, we aimed to understand the physical mechanism that enable PM superelasticity.

### 2.3 Nanotube Retraction Leads to Compositional Change of the Outer Membrane Segment

It seems likely that the extraordinary tGPMV deformability proceeds by the recruitment of the nanotubes into the outer membrane. Indeed, recruitment of membrane material from the nanotube could be directly visualized by fluorescence lifetime measurements (FLIM): FAST‐DiI lifetime is a measure of membrane composition and is related to the order parameters of the acyl chain over a wide range of membrane composition, but not to membrane curvature alone (Figure S1, Supporting Information; ref. [21]). Color coding for FAST‐DiI fluorescence lifetime immediately reveals two distinct populations of fluorescence lifetimes between the outer membrane segments and nanotubes (Figure 2c), indicating a stark contrast in composition and lipid order.

Next, we observed the fluorescence lifetime of the outer membrane segment upon aspiration of a tGPMV. We found that the fluorescence lifetime of the dye in the outer membrane segment shifts by a value of *Δ* toward the lifetime found in the nanotubes (Figure 2c). Control aspiration experiments on GPMV (Figure 2c) exhibit no such shift in lifetime (*Δ* ≈ 0). We also attempted to measure the corresponding shift in fluorescence lifetime in the nanotube network. Compared to the outer membrane segment, the resolution is limited by the lower and more heterogenous signal. On average, a small shift toward the outer membrane lifetime was obtained, which was not found to be significantly different from zero (Figure S2, Supporting Information). Thus, we speculate that the nanotube network acts as a large reservoir of constant membrane composition. Similar to constructing a tie line in a phase diagram using the lever‐rule, the exact partitioning behavior will depend on the relative area fractions of outer membrane segment and nanotubes.

In summary, these measurements represent direct evidence of mixing of the two membrane segments with tension. Essentially, we witness a continuous phase change of the outer membrane as a response to an increase in aspiration pressure and membrane tension.

### 2.4 Outer Membrane Segment and Nanotubes Have Distinct Membrane Compositions

The difference in composition between nanotubes and the outer membrane segment will be crucial for the interpretation of the tGPMV “superelastic” response and was corroborated by further experiments. Co‐staining with the dyes FAST‐DiI and DiD reveals segregation of the two lipid‐like molecules between the outer membrane segment and the highly curved nanotubes (Figure 2d). FAST‐DiI and DiD have similar molecular structure, except that FAST‐DiI exhibits a double bond in the acyl chain. Compared to this small chemical difference, a strikingly different partitioning of the two dyes is obtained. In fact, within the detection limit of our experimental setup, which approaches single‐molecule sensitivity, no fluorescence signal from DiD in the nanotube membrane segment is detected. Interestingly, the two dyes were shown to segregate during endocytosis in cell experiments,^[^ ^22^ ^]^ but dyes of similar acyl chain chemistry have shown neglectable sorting in synthetic vesicles before.^[^ ^23^ ^]^ Prompted by this finding, we investigated the nanotube composition using a series of typical PM fluorescent stains. The nanotube segment is rich in membrane protein as seen by NHS‐Alexa 647, which forms complexes with protein primary amines. Nanotubes were found negative for Transferrin marker, indicating absence of clathrin coats, and were also found negative for Annexin A5 binding. Nanotubes were positive for staining with Cholera toxin subunit B (CT‐B) (Figure 2e). These results are consistent with cylindrical morphology of the nanotubes (shown to reduce Annexin A5 binding affinity^[^ ^24^ ^]^), which, as shown in cells, are stained by nonclathrin mediated endocytosis marker CT‐B.^[^ ^10^, ^25^ ^]^ Taken together these results indicate strong partitioning of lipids and protein in the curvature field of the two membrane segments, effectively coupling membrane composition, phase state, and curvature.^[^ ^26^, ^27^, ^28^ ^]^ The partitioning of PM lipids between highly curved nanotubes and outer membrane segments is in agreement with predictions from molecular dynamics simulations informed by plasma membrane lipidomics,^[^ ^29^ ^]^ further corroborating our observations. In fact, we could have arrived at a similar conclusion without the need for aspiration experiments: upon osmotic deflation, the outer membrane segment starts to fluctuate with optically resolvable amplitudes, indicating a membrane tension in the entropic regime.^[^ ^6^ ^]^ This implies that the outer membrane is close to its preferred membrane curvature.^[^ ^30^ ^]^ Taking tGPMV diameter of about 5 µm yields *m*_outer_ < 1/5 µm^−1^ for the spontaneous curvature of the outer membrane, while the nanotubes have a spontaneous curvature of *m*_tubes_ > 5 µm^−1^ as implied by tube diameter below the optical resolution. Thus, tGPMVs must exhibit two membrane domains of distinct curvature and composition.

Combining all results, we see that when the plasma membrane is left to relax and equilibrate from cytoskeletal pining, it generates vesicles that consist of two membrane domains with remarkably different composition and curvature. Together with previous studies on the miscibility temperature of GPMVs^[^ ^5^ ^]^ and critical fluctuations,^[^ ^31^ ^]^ our studies complement the thermodynamic characterization of plasma membrane extracts.

### 2.5 Membrane Curvature and Domains Separation Act to Stabilize Area Reservoirs and Lead to Superelastic Response

To explain how the multicomponent nature of tGPMVs is responsible for their cell‐like elasticity, we have adopted a minimal working model based on the observation that nanotubes have considerably different composition with respect to the outer membrane (Figure 2d,e) and that the composition of the outer membrane changes upon pipette aspiration (Figure 2c). The high curvature prevents a fraction of the PM molecules to enter the nanotubes, so that they can provide a reservoir for only a subset of the species present in the whole membrane. In the Supporting Information, we show how, in general, the compositional shift of the outer membrane induced by aspiration generates restoring forces due to curvature–composition interactions. These forces emerging from unfavorable lipid–lipid interactions can easily oppose any suction pressure, and prevent the development of droplet‐like instabilities as observed in homogeneous tubulated GUVs.^[^ ^17^ ^]^ For the range of relative area changes studied here, the measured aspiration tension has a general structure

(1) $$\frac{\Delta PR_{p}\;}{2\left(1-R_{p}/R_{v}\right)}≃\Sigma_{\mathrm{app}}+K_{\mathrm{app}}\frac{\Delta A}{A_{0}}$$

where *A*_0_is the outer membrane area before aspiration, Δ*A* is the area increase due to suction, Σ_app_ is a tension term, which depends on the composition of both domains, and *K*_app_ is the “apparent” elastic modulus (see Equation (S27) in the Supporting Information). While in general *K*_app_ contains several contributions due to the different ways in which membrane composition can couple to curvature, we can show that, to explain the measured value of 3.1 ± 0.2 mN m^‐1^ (see Figure 2a), the leading term in the modulus has the simple form

(2) $$K_{\mathrm{app}}≃{\left(\Phi_{\mathrm{om}}-\Phi_{\mathrm{nt}}\right)}^{2}\frac{\partial^{2}e\left(\Phi_{\mathrm{om}}\right)}{\partial \Phi^{2}}$$

where Φ_om_ and Φ_nt_ are two order parameters characterizing the composition of the outer membrane and nanotubes, while *e*(Φ_om_) is the free energy density per unit area of a membrane with composition Φ_om_. Using mean‐field theory estimate for the energy, we calculate that a lipid–lipid interaction strength of the order of the thermal energy (see Equation (S41) in the Supporting Information) can reproduce the measured value of Σ_app_, consistently with previous experimental values.^[^ ^32^ ^]^ The simple structure of Equation (2) when compared with the dependency of Σ_app_ on the nanotube composition can explain why the observed trajectories in Figure 2a have the same slope but different intercept (see Equation (S25) in the Supporting Information).

In principle, there might be additional energetic contributions arising during aspiration, due to, e.g., scaffolding of proteins,^[^ ^33^ ^]^ line tension, the finite size of the nanotube network, crosslinking between the nanotubes, or changes in the elastic parameters of the membrane itself.^[^ ^34^ ^]^ All these effects would likely act to increase *K*_app_ further. Here, we have shown that the observed remixing of lipids between nanotube reservoir and the outer vesicle is sufficient to explain the magnitude of the measured apparent elastic response. Compared to vesicles without area‐reservoirs, the described effect has much lower energetic cost than tension‐induced changes to the preferred area per lipid. This distinguishes our finding from the conventional elasticity of simple lipid membranes, which has a mechanical, rather than chemical, origin and is similar to the stiff stretching response of GPMVs without nanotube network (Figure 2b). Another limit case of our model case are vesicles with nanotubes of the same composition as the outer segment (ΔΦ  =  0), in this case, *K*_app_ vanishes, consistently with previous experiments.^[^ ^17^ ^]^ We conclude that tGPMVs are able to withstand much larger deformations as their elastic response is not limited by the increase in mechanical membrane tension, but by the available lipid material in the nanotubes.

### 2.6 Bottom‐Up Construction of Superelastic Vesicles

**Figure 3:**
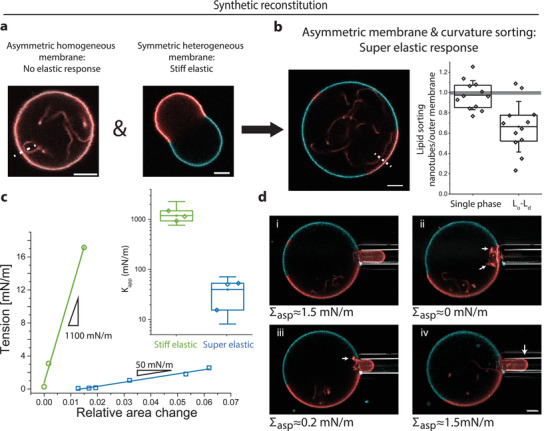
a) Tubulated homogenous DOPG:cholesterol GUV with asymmetric membrane and heterogenous symmetric DOPG:SM:cholesterol GUV. In the second image, cyan color shows TopFluor‐cholesterol (liquid ordered) and red DiIC:18 (liquid disordered). b) Asymmetric and heterogenous 3:5:2 DOPG:SM:cholesterol tGUV. The plot shows normalized membrane composition between outer liquid disordered membrane segment and nanotube (measured along the white dashed line) for single phase and *L*_o_–*L*_d_ phase separated GUVs. c) Micropipette aspiration data of nontubulated symmetric (green) and tubulated asymmetric (blue) DOPG:SM:cholesterol GUVs of the same overall composition. Inset: Apparent elastic constant *K*_app_ deduced from the area change with applied tension. Note the logarithmic scale. Each data point indicates an independent experiment. d) Reversibility of recruitment of nanotubes from phase separated GUV. Time series from (i)–(iv) where the intermediate aspiration at tension (iii) was equilibrated before suction pressure increases. All scale bars are 5 µm.

After we have identified the physics behind superelasticity in tGPMVs, we demonstrate that vesicles with similar size and elastic properties can be created in the lab de novo from synthetic lipids. In synthetic giant unilamellar vesicles (GUVs) nanotubes are generated by membrane spontaneous curvature, which is induced by membrane asymmetry.^[^ ^12^, ^35^, ^36^ ^]^ Following a method reported previously by us, membrane asymmetry is generated by sorting of the charged lipid DOPG (1,2‐Dioleoylphosphatidylglycerol) between membrane leaflets^[^ ^37^ ^]^ (Figure S3, Supporting Information). Upon osmotic deflation, DOPG:cholesterol GUVs form inward‐pointing nanotubes, adopting a similar vesicle morphology to tGPMVs. However, in contrast to tGPMVs, DOPG:cholesterol GUVs exhibit nanotubes of the same composition as that of the outer membrane segment (**Figure** **3**a,b—red data). Upon application of a small negative pressure difference, nanotubes in DOPG:cholesterol GUVs flow into the outer membrane at constant tension. As studied previously, constant membrane tension upon aspiration leads to a droplet‐like instability of the GUV in the glass‐capillary and no elastic response.^[^ ^17^ ^]^ This behavior is indeed the limit case of *K*_app_ → 0 studied here (see discussion of Equation (2)). To create an energetic cost for the mixing of nanotubes and outer membrane lipids, we introduce sphingomyelin (SM) as a third membrane lipid. Sphingomyelin tends to form a liquid ordered phase with cholesterol and segregates away from the liquid disordered DOPG‐rich phase, leading to lipid liquid–liquid phase separation.^[^ ^38^ ^]^ Proximity to the liquid–liquid miscibility gap was previously shown to enhance lipid sorting in pulling experiments of force induced nanotubes.^[^ ^34^, ^39^ ^]^ Indeed, spontaneous lipid curvature sorting could be visualized by dye‐labeled cholesterol and phospholipid analogues: in phase separated tGUVs, the dye intensity ratio between *L*_d_ segment of the outer membrane and *L*_d_ nanotube segment shows a compositional difference between the two membrane segments (Figure 3b). As expected, in control experiments on single‐phase GUVs lipid sorting is weak and the nanotubes appear to have the same membrane composition as the outer membrane segment (Figure 3b).

Consistent with our hypothesis, only in asymmetric phase‐separated tGUVs tension‐induced nanotube retraction ascribes a superelastic response: Aspirated DOPG:SM:cholesterol tGUVs appear over an order of magnitude softer and about three times more deformable compared to the bare symmetric DOPG:SM:chol GUVs without any area reservoirs (Figure 3c). Both types of GUVs have the same overall membrane composition, but exhibit a subtle nanoscopic structural difference, namely, in their membrane asymmetry. The deformations in tubulated GUVs were reversible, allowing for recruitment and reforming of nanotubes (Figure 3d).

## 3 Discussion

We found that structurally intact PM isolated from cells shows similar elastic properties to cells when probed by micropipette aspiration. We demonstrate that this is due to the presence of a network of lipid nanotubes with different composition, and compositional heterogeneity reminiscent of liquid–liquid coexisting phases. Plasma membrane extracts not only exhibit planar phase separated domains^[^ ^5^ ^]^ but are positioned to couple phase state and membrane curvature. While we believe that the high density of the tGPMV nanotube network is formed due to the unphysiological isolation procedure and loss of cytoskeletal pinning and tension, these nanotubes nonetheless bear similarities to the CLIC/GEEC nanotubes found in vivo. Similar to tGPMV nanotubes, cellular nanotubes were found positive for the membrane dye FAST‐DiI, negative for Transferrin marker and with a diameter of about 40 nm.^[^ ^10^ ^]^ CLIC/GEEC nanotubes were implicated previously in PM tension regulation,^[^ ^40^ ^]^ which is consistent with the tension magnitude leading to tGPMV nanotube retraction. We have shown that rebuilding tGPMV main features of nanotube formation, stabilized by membrane spontaneous curvature and lipid–lipid interactions, enables a bottom‐up synthetic reconstitution of superelastic tGUVs. Liquid–liquid phase separated membranes were widely studied in their phase behavior at various temperatures, showing only weak influence of mechanical tension.^[^ ^41^ ^]^ By introducing the coexistence of not only lipid domains, but also large differences in membrane domain curvature, we have obtained a novel synthetic membrane system, which, we anticipate, will exhibit rich isothermal phase behavior by tension changes. Typically lipid bilayer elastic response is linked to expansion of the bilayer/water interface from its preferred area, where the interfacial tension stems from the hydrophobicity of the lipid tails.^[^ ^42^ ^]^ Here, we show that a different type of elastic response can be engineered using nanotubular lipid domains that act as area reservoirs. The elastic response is now determined by two control parameters of domain composition and lipid–lipid interactions. Compared to the relatively constant lipid tail to water hydrophobicity, our mechanism allows for large deformations and precise control over the elastic response. In addition, the studied mechanism provides us with the ability to sort and reorganize lipids and proteins on plasma membrane mimetics by application of minute mechanical cues. In this way, superelastic membranes are bound to have a wide applicability in engineering of synthetic cells.

Compared to established superelasticity of phase‐changing metal alloys,^[^ ^43^ ^]^ tGPMV and their synthetic reconstitution allow for extensive 3D remodeling, demonstrating the remarkable features of membrane fluidity.

## 4 Experimental Section

### GPMV and tGPMV Generation and Isolation

PM blebbing was induced according to previously published protocols^[^ ^16^ ^]^ with modifications: U2OS cells were cultured in DMEM medium (10% FBS, 1% Penn‐Strep) to 70% confluency, washed three times with 5 mL phosphate buffered saline (PBS), incubated at 4 °C for 10 min in PBS (1 mL suspension was supplemented with 1 µL 10 mg mg^−1^ FAST‐DiI or DID (Thermofisher) in ethanol for experiments shown in Figures 1b and 2a–d), washed three times in buffer (10 × 10^−3^ m HEPES, 150 × 10^−3^ m NaCl, 2 × 10^−3^ m CaCl_2_, pH 7.4), and incubated for 1 h with 2 × 10^−3^ m NEM in buffer (1 mL). As indicated in the main text, in some experiments, tGPMV induction was suppressed by addition of 25 × 10^−3^ m PFA to the chemical NEM resulting in the formation of nontubulated GPMVs. PM blebbing induction with 2 × 10^−3^ m DTT and 25 × 10^−3^ m PFA in buffer yielded similar results, namely, GPMVs lacking a tubular network. Cells were incubated for an hour at 37 °C and GPMV/tGPMV were harvested. Here, special precaution was taken to minimize agitation of the flasks. Vesicles were isolated by single pipetting from slightly tilted T‐75 flasks yielding 1 mL supernatant. This procedure minimized contaminations from dead cells and other debris. After additional staining steps (see below), the harvested GPMVs were left to stand upright in an Eppendorf tube (1.5 mL volume) to let vesicles sediment by gravity for at least 30 min. A 100 µL sample collected from the bottom of the tube was then further analyzed within 12 h of sample isolation.

### General Sample Preparation

Coverslips were cleaned (rinsed with ethanol and Millipore water) and coated with bovine serum albumin (BSA) (incubated with 100 µL 1 mg mL^−1^ BSA for at least 30 min and washed with Millipore water). Between 30 and 50 µL of the tGPMV/GPMV solution was pipetted on the coverslip and sealed with a top cover glass separated by a spacer.

### Staining with Water Soluble Dyes

Water soluble dye and conjugated proteins were prepared according to the manufactures instructions and incubated for 20 min with tGPMV and GPMV solutions after isolation from the cell culture to the following final concentrations: 400 × 10^−6^ m Alexa Fluor 647 NHS‐Ester (Thermo Fisher), 2.5 × 10^−6^ m sulforhodamine B (Sigma), 16.7 × 10^−9^ m cholera toxin subunit B—Alexa Fluor 488 (Invitrogen), 130 × 10^−9^ m Human Transferrin—CF488A (Biotium), and 65 × 10^−9^ m annexin V—CF594 (Biotium).

### High‐Resolution Imaging

To obtain high‐resolution images of the tubular network, the diffusive nanotube motion was suppressed by protein crosslinking. The crosslinker PFA was added to tGPMVs after isolation (with NEM) from the cell culture. Importantly, crosslinking was only effective if PFA was supplemented with 0.17 × 10^−3^ m Triton X 100 (Sigma‐Aldrich) to increase membrane permeability. If the membrane was not labeled during plasma membrane isolation, 1 µL of 1 mg mL^−1^ solution in ethanol STED dye (ATTO 647N DOPE, Sigma‐Aldrich) or FAST‐DiI was added to 1 mL solution of isolated crosslinked tGPMVs. tGPMV was incubated for an hour at room temperature and imaged.

Image deconvolution was accomplished by the integrated deconvolution plugin (Huygens) in the Leica LAS X software. Automatic deconvolution settings were applied to slightly oversampled confocal images obtained using a 63×, NA 1.2 water immersion lens. STED microscopy was performed on an Abberior Instruments microscope with dye excitation at 640 nm and STED depletion at 775 nm using a pulsed laser source. STED alignment was accomplished using immobilized gold beads (Abberior), by adjusting the focus of the excitation beam into the center of the donut‐shaped depletion laser. Corrections for mismatches between the scattering mode and the fluorescence mode were achieved using TetraSpeck (ThermoFisher) beads of four colors. Crimson beads of 28 nm diameter were used to measure the resolution of STED, which was found to be about 35 nm.^[^ ^44^ ^]^

### FLIM Experiments

FLIM experiments were conducted on an Abberior Instruments microscope with pulsed 561 excitation. Fluorescent lifetime traces were analyzed using Becker & Hickl SPCM. The data were deconvoluted using the instrument response function (obtained by measurements of DASPI dye emission in methanol). Double‐exponential fits were performed on binned data within a membrane segment as indicated by the white rectangle in Figure 3 and Figure S2 in the Supporting Information, and the weighted average lifetime was reported. Fit was performed on the free parameters of amplitudes, lifetimes, and parameters “shift” and “scatter” using the SPCImage software. For the color‐coded image in Figure 2b, each pixel was fitted to a corresponding lifetime and the lifetime histogram competed over the whole image. The histogram was then smoothed with a window size of 50 ps.

### Image Quantification

Quantification of confocal images was performed using Fiji/ImageJ v. 2.0. For Figure 2e, the fluorescent signal in a circular section inside the tGPMV (radius 1 µm) and at the membrane (radius 0.1 µm) was averaged and the ratio was reported. For Figure 3b, confocal images were obtained on tubulated GUVs and a line profile was drawn across the liquid disordered outer membrane segment and a nanotube in proximity to the outer membrane segment. The confocal scanning direction was chosen to minimize polarization artifacts and only nanotubes roughly parallel to the outer membrane were considered. For each membrane segment, the peak intensity of the green and red fluorescent channels (Top flour‐Chol and DiIC:18) $I_{\mathrm{chol}}^{\mathrm{nt}},\;I_{\mathrm{chol}}^{\mathrm{om}},\;I_{\mathrm{DiI}}^{\mathrm{nt}},I_{\mathrm{DiI}}^{\mathrm{om}}$ were measured. The ratio between the green/red signals in the outer membrane and nanotube were then calculated as $\frac{I_{\mathrm{chol}}^{\mathrm{nt}}/I_{\mathrm{chol}}^{\mathrm{om}}}{I_{\mathrm{DiI}}^{\mathrm{nt}}/I_{\mathrm{DiI}}^{\mathrm{om}}}$. Values close to one indicate no sorting, and values below one indicate enrichment of the DiIC:18 dye relative to TopFluor‐Chol in the nanotubes. In cases where the membrane contour was poorly identified from the fluorescent images, the location of the outer membrane segment was estimated from phase contrast images.

### GUV Formation

GUVs were formed by electroformation from dried lipid stacks deposited on indium tin oxide (ITO)‐coated glasses as electrodes. As indicated, 8:2 DOPG:cholesterol or 3:5:2 DOPG:SM:cholesterol was deposited as a thin film (4 µL of 4 × 10^−3^ m lipid solution in chloroform spread on an area of about 1.5 × 3 cm^2^, 2 h drying at vacuum) on ITO glasses. ITO glasses were then assembled with a Teflon spacer to form a chamber of 2 mL volume that was filled with a buffered sucrose solution (17 mg mL^−1^ sucrose, 2 × 10^−3^ m HEPES pH 7.4, containing 1 × 10^−3^ m EDTA). Lipids were obtained from Avanti Polar Lipids and other chemicals were obtained from Sigma. In a protocol established before, by application of a low AC‐voltage during formation (640 mV_rms_, 10 Hz, 2 h, 60 °C), DOPG was distributed asymmetrically in between the leaflets.^[^ ^37^ ^]^ The lipid stocks for GUVs were doped with 0.1 mol% DiIC:18 (1,1′‐dioctadecyl‐3,3,3′,3′‐tetramethylindodicarbocyanine‐5,5′‐disulfonic acid, Thermofisher) and 0.5 mol% TopFluor‐Cholesterol (23‐(dipyrrometheneboron difluoride)‐24‐norcholesterol, Avanti Polar Lipids). DOPG asymmetry decayed over time: nanotubes mostly disappeared or widened to optically resolvable diameters during storage at room temperature overnight because of passive lipid flip‐flops equilibrating the membrane asymmetry. This population was then used in the “stiff elastic” experiments in Figure 3c. Before micropipette experiments, GUVs were diluted 1:10 in glucose solution (11 mg mL^−1^).

### Micropipette Experiments

Micropipettes were prepared from glass capillaries (World Precision Instruments Inc.) that were pulled using a pipette puller (Sutter Instruments, Novato, CA). Pipette tips were cut using a microforge (Narishige, Tokyo, Japan) to obtain smooth tips with inner diameter between 3 and 10 µm. Adhesion of the membrane to the pipette was prevented by incubation of the pipette tips in 1 mg mL^−1^ solution of casein or BSA (Sigma). A new pipette was used for the aspiration of each GUV. After the pipette was inserted into the observation chamber, the zero pressure across the pipette tip was attained and calibrated by watching the flow of small particles within the tip. The aspiration pressure was controlled through adjustments in the height of a connected reservoir mounted on a linear translational stage (M‐531.PD; Physik Instrumente, Germany). This setup^[^ ^17^ ^]^ allowed the pressure to increase up to 2 kPa with a resolution of 1 mPa. The pressure was changed by displacing the water reservoir at a speed of 0.01 mm s^−1^. At every step, the system was left to equilibrate for 2−3 min before any data were recorded. Images were analyzed using the ImageJ software. The area of the membrane forming a tongue in the pipette and large spherical segment (“outer membrane”) was directly measured from the membrane contour. The relative area change was calculated as $\frac{A-A_{0}}{A_{0}}$, where *A* is the measured vesicle area and *A*_0_ is the spherical outer membrane segmented before aspiration.

## Conflict of Interest

The authors declare no conflict of interest.

## Author contributions

J.S. and R.D. designed the project. T.B. performed micropipette aspiration experiments. F.S.C.L. performed dye quenching experiments. Z.Z. helped with STED experiments. J.S. performed all other experiments and initial model building. P.F. and R.L. developed the full theory. J.S., R.D. and P.F. wrote the manuscript. All authors discussed the results and commented on the manuscript.

## Supporting information

Supporting Information

## Data Availability

The data that support the findings of this study are available from the corresponding author upon reasonable request.
