# Supplementary material for: Superelasticity of Plasma‐ and Synthetic Membranes Resulting from Coupling of Membrane Asymmetry, Curvature, and Lipid Sorting
Source: Adv Sci (Weinh). 2021 Sep 26;8(21):2102109. doi: 10.1002/advs.202102109 (PMC8564416; doi:10.1002/advs.202102109)
Supplement: Supplementary file 1 — Supporting Information [file ADVS-8-2102109-s001.pdf]

© 2021 Wiley-VCH GmbH

ADVANCED SCIENCE
Open Access

Supporting Information

for *Adv. Sci.*, DOI: 10.1002/advs.202102109

Superelasticity of Plasma- and Synthetic Membranes Resulting from Coupling of Membrane Asymmetry, Curvature, and Lipid Sorting

Jan Steinkühler, Piermarco Fonda, Tripta Bhatia, Ziliang Zhao, Fernanda S. C. Leomil, Reinhard Lipowsky, and Rumiana Dimova**

Supplemental Information

Super-elasticity of plasma- and synthetic membranes resulting from coupling of membrane asymmetry, curvature and lipid sorting

Jan Steinkühler[1,2*], Piermarco Fonda[1], Tripta Bhatia[1,3], Ziliang Zhao[1], Fernanda S. C. Leomil[1,4], Reinhard Lipowsky[1], Rumiana Dimova[1*]

[1] Theory and Bio-Systems, Max Planck Institute of Colloids and Interfaces, Science Park Golm, 14424 Potsdam, Germany

[2] Current address: Department of Biomedical Engineering, Northwestern University, Evanston, IL 60657, USA

[3] Department of Physical Sciences, Indian Institute of Science Education and Research Mohali, Sector 81, Knowledge City, S. A. S. Nagar, Manauli 140306, India

[4] Departamento de Biofísica, Universidade Federal de São Paulo, 043039-032, Brazil

Supplemental Figures

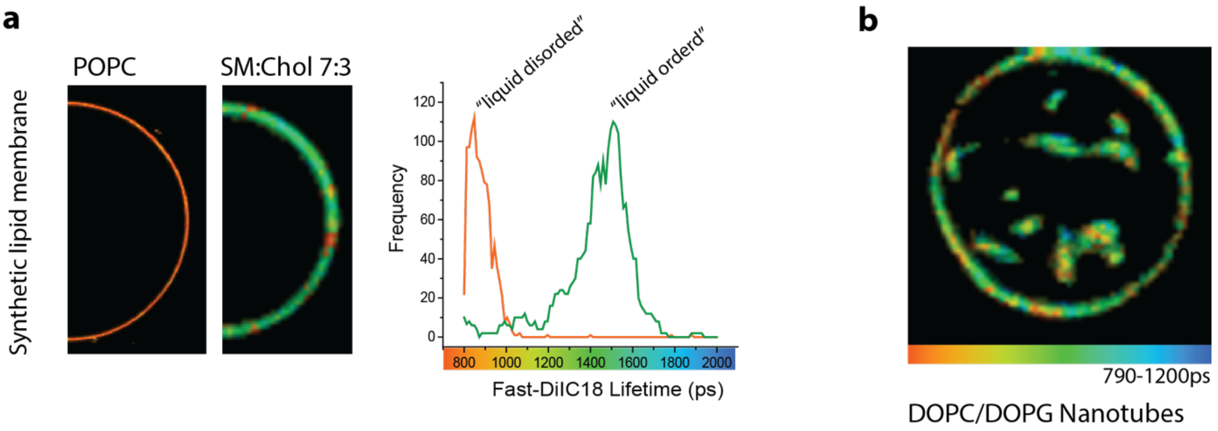

Fig. S1 – Control FLIM measurements. a) Fast-DiI fluorescent lifetime is sensitive to membrane order in synthetic POPC "liquid-discorded" (left) and liquid-ordered egg sphingomyelin:cholestetrol (right) 7:3 membranes. GUVs were prepared at the indicated lipid concentrations as described in the main text with 0.1 mol% Fast-DiI dye. Both signals show representative GUVs from separate experiments of each composition. Color code shows FLIM lifetime. b) In homogenous DOPC:DOPG GUVs (see main text method) which exhibit nanotubes of the same composition as the outer membrane segment, Fast-DiI does not have a significant shift in fluorescent lifetime, indicating that Fast-DiI is not sensitive to membrane curvature alone.

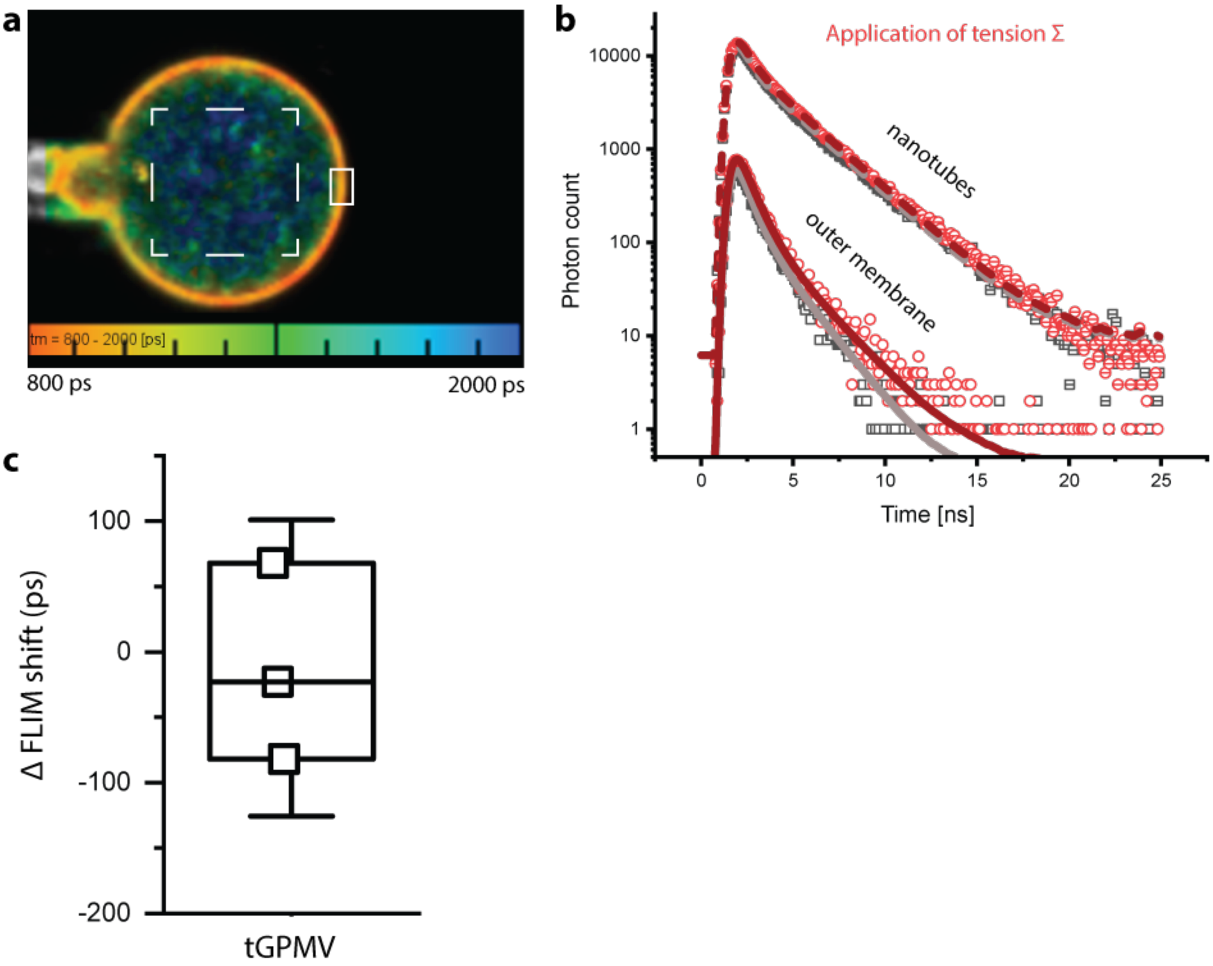

Fig S2 – Quantification of the negligible tension-induced shift of the average fluorescence lifetime of the nanotube network in tGPMVs. a) White square and rectangle indicate segments used for binning of nanotube lifetime data and subsequent fitting. Color code shows fluorescence lifetime and demonstrates the large variability measured in the nanotube network. b) Example traces obtained from binned signal (square/rectangle in panel a). Solid curves (data for outer membrane) and dashed curves (nanotubes) are 2-component exponential decay fits. Traces in red/dark red correspond to signal from the same segment after aspiration with a small tension Σ. In this example, the tension-induced shift in outer membrane fluorescence lifetime corresponds to about $\Delta \approx 93$ ps, while it was not resolvable for the nanotubes. c) Analysis of the shift in the average nanotube lifetime after application of a small tension difference 0.7 ± 0.3 mN/m for n=3 repeats.

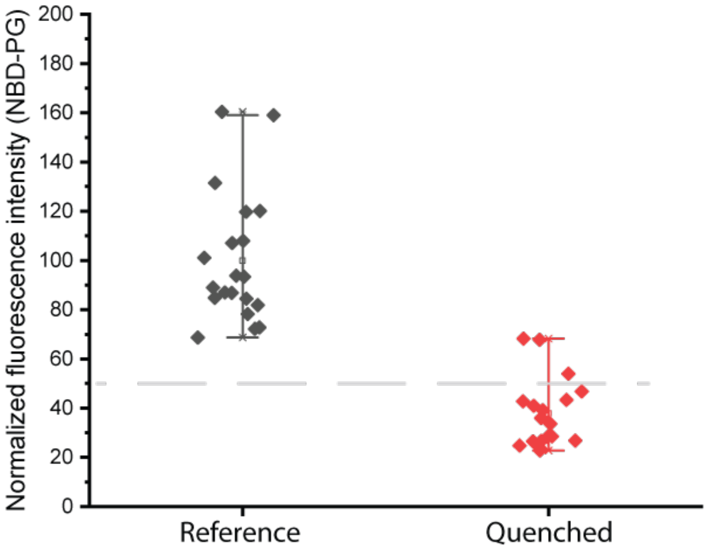

Fig S3 – Results of quenching 3/5/2 DOPG/eggSM/chol GUVs doped with tail-labeled NBD-PG. The reference signal of unquenched vesicles was normalized to 100 intensities units. Addition of a membrane impermeable NBD quenching agent to the GUV external solution shifted the fluorescent intensities to a mean of 38 intensity units (see SI Methods for details). These results indicate accumulation of DOPG in the outer bilayer leaflet during the GUV formation procedure and establishes the molecular basis for the spontaneous curvature of tGUVs. Gray dashed line indicates 50 intensity units. For details of the assay and sorting of DOPG see (Steinkühler et al., 2018).

Supplemental Methods

Quenching Experiments

For each experiment, we first prepared a fresh 100 mM stock solution of sodium dithionite (Sigma) in 1M Tris-HCl buffer. 3/5/2 DOPG/eggSM/chol GUV with 1 mol% NBD-PG (1-oleoyl-2-[12-[(7-nitro-2-1,3-benzoxadiazol-4-yl)amino]dodecanoyl]-sn-glycero-3-[phospho-rac-(1-glycerol)] (ammonium salt) (Avanti Polar Lipids)) (for other details see main text methods) were grown using electro-formation in 200 mM sucrose HEPES pH 7.4 + 0.1 mM EDTA buffer, similar to the protocol in Ref. (Steinkühler et al., 2018). Then, 1.5 µL of this quenching buffer was pipetted into 58.5 µL of vesicle suspension in 200 mM glucose buffer (40 µL of glucose + 18.5 µL of GUV). The solution was gently stirred to ensure homogenous distribution of the quenching agent. The vesicles were then incubated for 5 minutes and consecutively diluted in 200 mM glucose buffer (to a final volume of 300 µL). From this final mixture, 60 µL was used for observation. For each population (non-quenched and quenched), at least 18 GUVs were imaged at the equatorial plane. The membrane intensity was assessed from the average of 4 peak maxima from the line profiles, drawn in the 4 brightest regions of the membrane.

Steinkühler, J., De Tillieux, P., Knorr, R. L., Lipowsky, R., & Dimova, R. (2018). Charged giant unilamellar vesicles prepared by electroformation exhibit nanotubes and transbilayer lipid asymmetry. *Scientific Reports*. https://doi.org/10.1038/s41598-018-30286-z

Estimation of the chemically-induced super-elastic coefficient

Even though GPMV membranes are complex mixtures of several components, the essential mechanism through which the aspiration-induced mixing of differently curved domains generates the super-elastic response can be captured by a simple binary model. Therefore, for simplicity, we treat the tGPMV membrane as a two-component fluid consisting of two generic lipids species A and B.

The membrane is stable and there is no significant lipid exchange with the solvent, so both individual lipid numbers N^A and N^B are conserved quantities. We define the global membrane composition as

$$\Phi = \frac{N^A}{N^A + N^B} \,, \tag{S1}$$

which quantifies the relative number of A lipids in the membrane. The relative number of B lipids is given by $1 - \Phi$.

After tubulation, the tGPMV is at equilibrium with its surroundings and comprises primarily two environments: the nanotubes and the outer membrane domains. We label quantities relative to each of these domains respectively with nt and om. The two domains have different sizes, which we quantify via their areas $\mathcal{A}_{\mathrm{nt}}$ and $\mathcal{A}_{\mathrm{om}}$ and enclosed volumes V_{nt} and V_{om}. We assume that both the total area $\mathcal{A}_{\mathrm{tot}}$ and the total volume V_{tot} of the tGPMV are fixed quantities at equilibrium. Therefore, the relationships

$$\mathcal{A}_{\mathrm{tot}} = \mathcal{A}_{\mathrm{nt}} + \mathcal{A}_{\mathrm{om}} \,, \qquad V_{\mathrm{tot}} = V_{\mathrm{om}} - V_{\mathrm{nt}} \,, \tag{S2}$$

must hold. Note that because the nanotubes are inside the tGPMV, the volume V_{nt} must be *subtracted* from the outer segment one, so that $V_{\mathrm{tot}} < V_{\mathrm{om}}$. We also assume, for simplicity, that the average area per lipid, a, is the same for both species and is not changed significantly during aspiration. Similarly to Eq. (S1) we define the relative area fraction occupied by the outer membrane domain

$$y = \frac{\mathcal{A}_{\mathrm{om}}}{\mathcal{A}_{\mathrm{nt}} + \mathcal{A}_{\mathrm{om}}} \,, \tag{S3}$$

so that the nanotubes occupy an area $(1 - y)\mathcal{A}_{\mathrm{nt}}$.

We also need to introduce the local composition variables

$$\Phi_{\mathrm{nt}} = \frac{N^A_{\mathrm{nt}}}{N^A_{\mathrm{nt}} + N^B_{\mathrm{nt}}} \,, \qquad \Phi_{\mathrm{om}} = \frac{N^A_{\mathrm{om}}}{N^A_{\mathrm{om}} + N^B_{\mathrm{om}}} \,, \tag{S4}$$

which give the relative amount of A lipids in the nanotubes and the outer membrane. These compositions must match the global lipid fraction Eq. (S1) when weighted with the respective membrane area. By combining Eq. (S3) and Eq. (S4) together with $N^A = N^A_{\mathrm{nt}} + N^A_{\mathrm{om}}$ and $N^B = N^B_{\mathrm{nt}} + N^B_{\mathrm{om}}$ we obtain the further constraint

$$\mathcal{A}_{\mathrm{nt}}\Phi_{\mathrm{nt}} + \mathcal{A}_{\mathrm{om}}\Phi_{\mathrm{om}} = \mathcal{A}_{\mathrm{tot}}\Phi \,. \tag{S5}$$

Note that this expression entangles Φ_{om}, Φ_{nt} and the domain areas: a generic shape change of a domain affects its chemical nature.

The general form of the free energy

The free energy of the system is an extensive quantity, so we can decompose it as

$$F = F_{\mathrm{nt}} + F_{\mathrm{om}} \,, \tag{S6}$$

where $F_{\mathrm{nt,om}}$ are the local free energies of the respective domains. In principle, we should also include a line term quantifying the energy of the domain boundaries where the composition smoothly interpolates between Φ_{om} and Φ_{nt}. Since the length of this interface-like portion is expected to be small and not to change significantly during aspiration, we will ignore such a contribution in the following.

In general, both terms in Eq. (S6) can be written as surface integrals of local densities, which must depend on local intensive variables:

$$F_{\mathrm{nt}} = \int_{\mathcal{A}_{\mathrm{nt}}} \mathrm{d}A f(\Phi_{\mathrm{nt}}, M_{\mathrm{nt}}) \,, \qquad F_{\mathrm{om}} = \int_{\mathcal{A}_{\mathrm{om}}} \mathrm{d}A f(\Phi_{\mathrm{om}}, M_{\mathrm{om}}) \,, \tag{S7}$$

where $M_{\rm nt}$ and $M_{\rm om}$ are the local mean curvatures of the membrane[1]. For the explicit dependence of f on the geometry, we choose a generalised Canham-Helfrich density

$$f(\Phi, M) = e(\Phi) + 2\kappa(\Phi)\,(M - m(\Phi))^2 \ , \tag{S8}$$

where $e(\Phi)$ is the chemical free energy density per unit area, which, in general, will contain both internal and entropic (i.e. temperature dependent) contributions, while $\kappa(\Phi)$ and $m(\Phi)$ are respectively the composition-dependent bending modulus and spontaneous curvature.

It is precisely the coupling terms in Eq. (S8) between Φ and M that allow, at equilibrium, the two membrane domains to sustain different compositions, i.e. to have $\Phi_{\rm om} \neq \Phi_{\rm nt}$. Conversely, for homogeneous vesicles where $\Phi_{\rm om} = \Phi_{\rm nt} = \Phi$ (see Eq. (S5)), the free energy Eq. (S6) would reduce to the usual spontaneous curvature model of lipid membranes.

The equilibrium conditions for the tGPMV can be obtained by minimizing Eq. (S7) with respect to compositions and geometric variables. However, note that the variables entering Eq. (S6) are not all independent from each other: the global membrane composition, the total area and the total volume are all fixed quantities (see Eq. (S2) and Eq. (S5)). Finding minima of Eq. (S6) is thus a constrained minimisation problem which can be solved with the aid of Lagrange multipliers. We thus must consider the generalised free energy

$$G = G_{\rm nt} + G_{\rm om} \ , \tag{S9}$$

with

$$G_{\rm nt} = F_{\rm nt} + (\Sigma - \lambda\Phi_{\rm nt})\,\mathcal{A}_{\rm nt} + \Delta P V_{\rm nt} \ , \tag{S10a}$$

$$G_{\rm om} = F_{\rm om} + (\Sigma - \lambda\Phi_{\rm om})\,\mathcal{A}_{\rm om} - \Delta P V_{\rm om} \ , \tag{S10b}$$

where λ is the Lagrange multiplier enforcing Eq. (S5), Σ is the Lagrange multiplier enforcing the total area to be $\mathcal{A}_{\rm tot}$ and $\Delta P = P_{\rm in} - P_2$ is the pressure differential between the inside and the outside of the tGPMV.

Equilibrium configurations are those that satisfy $\delta G = 0$, where variations can be carried out independently for variables relative to each domain. These derivatives can be of two kinds: with respect to the chemical compositions or with respect to membrane shape, and will produce two sets of equations, to which we refer respectively as "chemical" and "mechanical" equilibrium conditions.

Before aspiration, the solution of these equations determines the equilibrium values $\Phi_{\rm nt}$ and $\Phi_{\rm om}$. Because of the composition-curvature couplings in Eq. (S8), we expect these to be different from each other. It is then useful to introduce the composition difference

$$\Delta\Phi = \Phi_{\rm nt} - \Phi_{\rm om} \ , \tag{S11}$$

which vanishes only for homogeneous vesicles.

Lipid and area recruitment upon aspiration

We model the tGPMV aspiration process as shown in Fig. S4. As the outer membrane is exposed to two different external pressures (denoted P_1 and P_2), its shape deviates significantly from a sphere. We capture the essential features of the tGPMV geometry as shown in Fig. S4b: the outer membrane segment consists of the union a spherical outer segment of radius $R_{\rm v}$ with a cylindrical portion inside the pipette of radius $R_{\rm p}$ and length $L_{\rm p}$ ending on a tongue-like spherical cap[2] of radius $R_{\rm t}$. At the initial aspiration stage there is no cylindrical segment (i.e. $L_{\rm p} = 0$) and the tongue radius decreases from $R_{\rm t} = R_{\rm om}$ (the original outer sphere radius) towards its minimum value $R_{\rm t} = R_{\rm p}$. In homogeneous tubulated vesicles it is precisely at this point that the droplet-like instability develops, as the suction pressure crosses a critical threshold and the whole vesicle suddenly flows inside the pipette [2]. In the present case, however, such instability is absent and the vesicle can sustain larger pressures so to have $L_{\rm p} > 0$ while maintaining $R_{\rm t} = R_{\rm p}$.

To avoid confusion with quantities referring to the non-aspirated tGPMV we will denote any variable relative to the aspirated vesicle with a prime $'$, so that e.g. $\mathcal{A}'_{\rm om}$ refers to the area of the aspirated vesicle.

Since $\mathcal{A}'_{\rm om} > \mathcal{A}_{\rm om}$, we define the area difference $\Delta\mathcal{A} = \mathcal{A}'_{\rm om} - \mathcal{A}_{\rm om} > 0$. From Fig. 2a we see that the super-elastic response happens for relative area changes, $\Delta\mathcal{A}/\mathcal{A}_{\rm om}$, in the range of $5 - 25\%$. Using the surface parametrization sketched in Fig. S4b, we can calculate exactly every geometrical aspects of the aspiration process, including $\Delta\mathcal{A}$. At the initial stage of the aspiration we have $L_{\rm p} = 0$ and the area increase is due only to the decrease of the tongue radius towards $R_{\rm p}$. Fig. S4c

[1] A completely general energy density would also depend on the local Gaussian curvature K. However, for simplicity and since linear terms in K are topological, we neglect such contributions here.

[2] The shape of the tongue cannot be exactly spherical [1], although this would lead to only minor corrections to Eq. (S23).

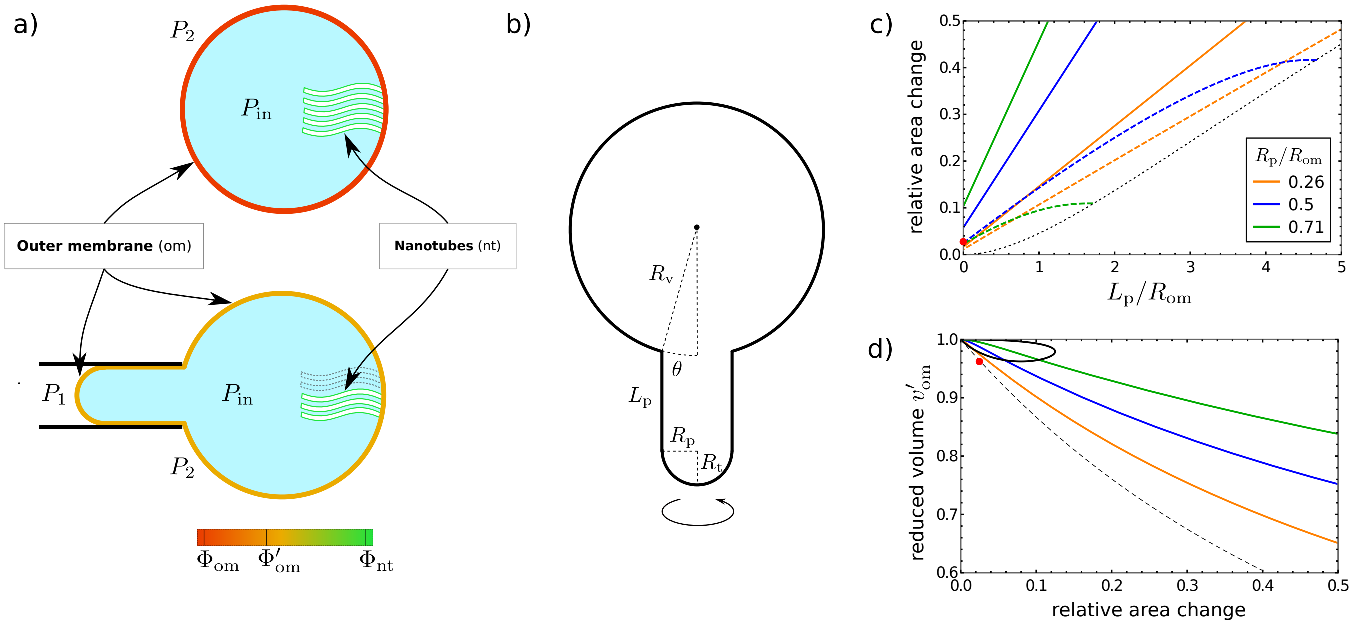

Figure S4: **a)** Cartoon showing the effect of aspiration on tGPMV shape and composition. *Top:* before aspiration, the tGPMV comprises two domains: an outer sphere of radius $R_{\rm om}$ and composition $\Phi_{\rm om}$ (red), and the nanotubes of cross-sectional radius $R_{\rm nt}$ and composition $\Phi_{\rm nt}$ (green). The tubes are inward pointing, so $V_{\rm tot} < V_{\rm om}$. *Bottom:* during aspiration new lipids are recruited from nanotubes (dashed outline) so that the outer segment changes shape but also composition, shifting to a value $\Phi'_{\rm om}$ (orange), closer to $\Phi_{\rm nt}$. **b)** The geometry of the outer segment during aspiration is well approximated by the union of two spherical caps and a cylindrical segment. The angle θ subtends a cone such that $R_{\rm p} = \sin\theta R_{\rm v}$. Initial aspiration has $L_{\rm p} = 0$ and $R_{\rm t} > R_{\rm p}$, until when $R_{\rm t} = R_{\rm p}$ and $L_{\rm p}$ starts increasing. **c)** Relative area increase $\Delta\mathcal{A}/\mathcal{A}_{\rm om}$ for the shape shown in **b** as a function of the aspirated length, from Eq. (S17). We model this aspiration either at constant volume (dashed lines) or at constant outer sphere radius $R_{\rm v} = R_{\rm om}$ (solid lines). The colours refer to different relative pipette sizes as shown in the legend. The dotted line is the maximal $L_{\rm p}$ for constant volume aspiration, beyond which the tGPMV is entirely suctioned. **d)** Reduced volume $v'_{\rm om} = 6\sqrt{\pi}V'_{\rm om}(\mathcal{A}'_{\rm om})^{-3/2}$ as a function of area increase, with the same colour coding as in **c**: constant $R_{\rm v} = R_{\rm om}$ aspirations (solid lines) discriminate between different $R_{\rm p}$, while constant volume aspirations collapse on the single dashed curve $(\Delta\mathcal{A}/\mathcal{A}_{\rm om})^{-3/2}$. The solid black line shows the boundary between the initial aspiration regime with $R_{\rm t} > R_{\rm p}$ and the suction with $L_{\rm p} \neq 0$. The red dot shows the maximum achievable $\Delta\mathcal{A}/\mathcal{A}_{\rm om}$ with $L_{\rm p} = 0$ for constant volume aspirations.

and Fig. S4d show how this initial phase is not relevant for the experimentally measured area changes, as for a wide range of relative pipette sizes $R_{\rm p}/R_{\rm om}$ the maximal increases at this stage are always below $\sim 5\%$. Dashed and continuous lines in these plots refer to two different assumptions on the outer membrane volume $V'_{\rm om}$ during aspiration: the former refer to constant outer radius deformations, i.e. $R_{\rm v} = R_{\rm om}$ is kept constant while $V'_{\rm om}$ increases; the latter refers to constant volume aspirations where $V'_{\rm om} = V_{\rm om}$ is kept constant and the outer sphere radius decreases, $R_{\rm v} < R_{\rm om}$. Experimentally it is hard to distinguish between these two limiting cases: on the one hand, the outer membrane segment radius seems to be constant within optical resolution, while on the other one would expect no significant volume change due to smallness of $V_{\rm nt} \propto R^2_{\rm nt}$. Likely, the experimental setup lies somewhere in between these two limits. Regardless, Fig. S4c and Fig. S4d clearly show that we can ignore the initial stage of aspiration and focus on the later stage with $L_{\rm p} > 0$ and $R_{\rm t} = R_{\rm p}$, as we shall do in the following.

Given the measured low value of the elastic modulus, we can safely assume that the average area per lipid a is also constant, which otherwise would lead to a significantly stiffer elastic response, as is the case for non-tubulated vesicles (see Figs. 2b and 3c). This means that the observed $\Delta\mathcal{A}$ is entirely due to lipid recruitment from the nanotubes, and thus represents an *area transfer* between domains. From this, it follows that the the total tGPMV area is constant during aspiration, and to every area increase of the $\rm om$ domain there must be a corresponding area decrease of the $\rm nt$ domain, namely

$$\mathcal{A}_{\rm nt} \rightarrow \mathcal{A}'_{\rm nt} = \mathcal{A}_{\rm nt} - \Delta\mathcal{A}\,, \tag{S12a}$$

$$\mathcal{A}_{\rm om} \rightarrow \mathcal{A}'_{\rm om} = \mathcal{A}_{\rm om} + \Delta\mathcal{A}\,, \tag{S12b}$$

so that $\mathcal{A}_{\rm tot}$ remains unchanged. Due to the multi-component nature of the membrane, this area transfer implies that the composition of outer membrane can change during aspiration, as shown in Fig. 2c. If lipid recruitment from the nanotubes proceeds uniformly, i.e. the amount of A and B lipids transferred from the nanotubes to the outer membrane is uniquely determined by $\Phi_{\rm nt}$, the recruited area will contain $2\Delta\mathcal{A}\Phi_{\rm nt}/a$ type A lipids and $2\Delta\mathcal{A}(1-\Phi_{\rm nt})/a$ type B lipids. We can drop this assumption at the expense of introducing a further variable quantifying the relative mole fraction $\Phi_{\Delta\mathcal{A}}$ in the transferred area $\Delta\mathcal{A}$. This could explain the shift in tubes composition observed during aspiration (see the shift of the FAST-DiI fluorescence lifetime distribution in Fig. 2c of the main text), but it is not necessary for the sake of the argument presented here. For the time being, we thus take $\Phi_{\Delta\mathcal{A}} = \Phi_{\rm nt}$ as a simplifying assumption.

It then follows that compositions change as

$$\Phi_{\rm nt} \rightarrow {\Phi_{\rm nt}}' = \Phi_{\rm nt}\,, \tag{S13a}$$

$$\Phi_{\rm om} \rightarrow {\Phi_{\rm om}}' = \Phi_{\rm om} + \Delta\Phi\frac{\Delta\mathcal{A}}{\mathcal{A}'_{\rm om}}\,, \tag{S13b}$$

where $\Delta\Phi = \Phi_{\rm nt} - \Phi_{\rm om}$ is the pre-aspiration equilibrium difference defined in Eq. (S11). the value of $\Phi_{\rm om}$ is pushed towards $\Phi_{\rm nt}$ (see Fig. S4a), and Eq. (S13b) clearly shows how the aspiration process entangles a *geometric* variation to a change in the *chemical composition* of the membrane. The outer membrane local curvature $M'_{\rm om}$ of the aspirated tGPMV is no longer constant, but rather a piece-wise constant function. Because of the curvature-composition couplings one would expect also $\Phi'_{\rm om}$ to be a piece-wise constant function over the three portions of the aspirated tGPMV membrane as well, so that Eq. (S13b) would quantify only an average compositional shift. In the experiments, however, no significant inhomogeneity in the outer segment has been observed. We can explain this by noting that the nanotubes typical curvature $M_{\rm nt} = 1/2R_{\rm nt}$ is at least two orders of magnitude larger than any characteristic curvature of the outer membrane (either $1/R_{\rm om}$, $1/R_{\rm v}$ or $1/R_{\rm p}$). We infer that the coupling mechanism inducing $\Delta\Phi \neq 0$ is too weak to generate a measurable inhomogeneity in the different portions of the outer membrane. We thus consider both the tongue and the external spherical cap being formed by a uniform membrane of compositions $\Phi'_{\rm om}$.

In order to link the applied pressure to geometric and compositional changes, we must minimise the free energy. Note that now, because we are considering $\Phi_{\rm nt}$ a fixed quantity, we must rewrite the constraint Eq. (S5) accordingly. Using Eq. (S13) and Eq. (S12) we have

$$\mathcal{A}_{\rm tot}\Phi = \mathcal{A}'_{\rm nt}\Phi'_{\rm nt} + \mathcal{A}'_{\rm om}\Phi'_{\rm om} = \mathcal{A}'_{\rm om}(\Phi'_{\rm om} - \Phi_{\rm nt}) + \mathcal{A}_{\rm tot}\Phi_{\rm nt}\,, \tag{S14}$$

where the last term in the right-hand side is a constant. At this point, we can ignore the nanotube domain and focus on the outer membrane segment. We thus consider only the second term in Eq. (S9):

$$G_{\rm om} = F_{\rm om} + \left(\Sigma - \lambda(\Phi'_{\rm om} - \Phi_{\rm nt})\right)\mathcal{A}'_{\rm om} - \sum_i (P_{\rm in} - P_i)V'_{{\rm om},i}\,, \tag{S15}$$

where

$$F_{\rm om} = \sum_i \mathcal{A}'_{{\rm om},i}\, f(\Phi'_{\rm om}, M'_{{\rm om},i})\,, \tag{S16}$$

and the sums span over the three constant curvature portions of the outer membrane domain, with $\mathcal{A}'_{\mathrm{om},i}$ and $V'_{\mathrm{om},i}$ being their respective areas and volumes such that $\sum_i \mathcal{A}'_{\mathrm{om},i} = \mathcal{A}'_{\mathrm{om}}$ and $\sum_i V'_{\mathrm{om},i} = V'_{\mathrm{om}}$. P_i are the external pressures to which each portion is subject to, while there is a single composition Φ'_{om} over the whole domain. In Eq. (S15), the Lagrange multiplier Σ enforces constraint Eq. (S12b) while λ enforces Eq. (S14).

Specifically, the areas and curvatures of these portions are, past the initial aspiration stage, respectively $1/R_{\mathrm{v}}$ and $2\pi R_{\mathrm{v}}(1+\cos\theta)$ on the outer spherical segment, $1/2R_{\mathrm{p}}$ and $2\pi L_{\mathrm{p}} R_{\mathrm{p}}$ on the cylinder and $1/R_{\mathrm{p}}$ and $2\pi R_{\mathrm{p}}$ on the tongue ($\theta = \arcsin R_{\mathrm{p}}/R_{\mathrm{v}}$ is the subtended angle of the pipette on the outer spherical segment). Using the surface parametrization of the aspirated outer membrane shown in Fig. S4b, the expression for the whole outer membrane domain area is

$$\mathcal{A}'_{\mathrm{om}} = 2\pi R_{\mathrm{v}}^2 \left(1 + \cos\theta + \sin^2\theta + \frac{L_{\mathrm{p}}}{R_{\mathrm{v}}}\sin\theta\right) , \tag{S17}$$

so that the relative area increase is

$$\frac{\Delta\mathcal{A}}{\mathcal{A}_{\mathrm{om}}} = \frac{R_{\mathrm{v}}^2}{2R_{\mathrm{om}}^2}\left(1 + \cos\theta + \sin^2\theta + \frac{L_{\mathrm{p}}}{R_{\mathrm{v}}}\sin\theta - 2\frac{R_{\mathrm{om}}^2}{R_{\mathrm{v}}^2}\right) . \tag{S18}$$

While this expression formally depends on two geometric ratios, $R_{\mathrm{om}}/R_{\mathrm{v}}$ and $L_{\mathrm{p}}/R_{\mathrm{v}}$, $\Delta\mathcal{A}$ truly depends on a single degree of freedom (e.g. the length L_{p}), since we still need to fix whether V_{om} or R_{om} is kept constant during aspiration. These different choices lead to two distinct expressions for $\Delta\mathcal{A}$ as a function of L_{p}, as shown respectively by the dashed and solid lines in Fig. S4c.

With the explicit form Eq. (S15) we can now carry out variations with respect to composition and shape on each portions of the outer membrane domain.

Chemical equilibrium Taking a derivative with respect to Φ'_{om} of G_{om} in Eq. (S15) leads to

$$\frac{1}{\mathcal{A}'_{\mathrm{om}}}\sum_i \mathcal{A}'_{\mathrm{om},i}\left.\frac{\partial f}{\partial\Phi}\right|_{\Phi=\Phi'_{\mathrm{om}},M=M'_{\mathrm{om},i}} = \lambda , \tag{S19}$$

which for the Canham-Helfrich density Eq. (S8) becomes

$$\partial_\Phi\left(e(\Phi'_{\mathrm{om}}) + 2\kappa(\Phi'_{\mathrm{nt}})m(\Phi'_{\mathrm{nt}})^2\right) + \frac{h_\kappa}{\mathcal{A}'_{\mathrm{om}}}\partial_\Phi\kappa(\Phi'_{\mathrm{om}}) - \frac{R_{\mathrm{v}} h_m}{\mathcal{A}'_{\mathrm{om}}}\partial_\Phi(\kappa(\Phi'_{\mathrm{om}})m(\Phi'_{\mathrm{om}})) = \lambda , \tag{S20}$$

where $\mathcal{A}'_{\mathrm{om}}$ is given by Eq. (S17) and we defined the two dimensionless auxiliary functions

$$h_\kappa(\theta, L_{\mathrm{p}}/R_{\mathrm{v}}) = 4\pi\left(2 + \cos\theta + \frac{L_{\mathrm{p}}}{4R_{\mathrm{v}}\sin\theta}\right) , \tag{S21a}$$

$$h_m(\theta, L_{\mathrm{p}}/R_{\mathrm{v}}) = 8\pi\left(1 + \cos\theta + \sin\theta + \frac{L_{\mathrm{p}}}{2R_{\mathrm{v}}}\right) . \tag{S21b}$$

Mechanical equilibrium The cylindrical part of the outer membrane is in direct contact with the pipette wall which, being a solid, can exert an arbitrary reaction pressure P_{wall} on the membrane, counterbalancing any mechanical force. Therefore this portion will not provide any physically relevant information about the the suction process and we can focus on the two other parts of the domain: the outer segment and the tongue. Since both are spherical caps, their shape equations are

$$P_{\mathrm{in}} - P_2 = 2\frac{\Sigma + e(\Phi'_{\mathrm{om}}) - \lambda(\Phi'_{\mathrm{om}} - \Phi_{\mathrm{nt}}) + 2\kappa(\Phi'_{\mathrm{om}})m(\Phi'_{\mathrm{om}})^2}{R_{\mathrm{v}}} - 4\frac{\kappa(\Phi'_{\mathrm{om}})m(\Phi'_{\mathrm{om}})}{R_{\mathrm{v}}^2} , \tag{S22a}$$

$$P_{\mathrm{in}} - P_1 = 2\frac{\Sigma + e(\Phi'_{\mathrm{om}}) - \lambda(\Phi'_{\mathrm{om}} - \Phi_{\mathrm{nt}}) + 2\kappa(\Phi'_{\mathrm{om}})m(\Phi'_{\mathrm{om}})^2}{R_{\mathrm{p}}} - 4\frac{\kappa(\Phi'_{\mathrm{om}})m(\Phi'_{\mathrm{om}})}{R_{\mathrm{p}}^2} , \tag{S22b}$$

which can be combined into a single equation

$$\frac{\Delta P}{2\left(\frac{1}{R_{\mathrm{p}}} - \frac{1}{R_{\mathrm{v}}}\right)} = \Sigma + e(\Phi'_{\mathrm{om}}) - \lambda(\Phi'_{\mathrm{om}} - \Phi_{\mathrm{nt}}) + 2\kappa(\Phi'_{\mathrm{om}})m(\Phi'_{\mathrm{om}})^2 - 2\kappa(\Phi'_{\mathrm{om}})m(\Phi'_{\mathrm{om}})\left(\frac{1}{R_{\mathrm{p}}} + \frac{1}{R_{\mathrm{v}}}\right) , \tag{S23}$$

where $\Delta P = P_2 - P_1$ is the difference between the pressure inside the pipette and the external environment. This equation generalises the well-known Laplace relation for homogeneous vesicles [3], as the Lagrange multiplier λ depends non-trivially on the geometric degrees of freedom of the vesicle via Eq. (S20). One recovers the standard result by setting $\lambda = 0$ and removing any $\Phi'_{\rm om}$ dependence.

Solving for λ in Eq. (S20) and substituting back into Eq. (S23) leads to

$$\frac{\Delta P}{2\left(\frac{1}{R_{\rm p}} - \frac{1}{R_{\rm v}}\right)} = \hat{\Sigma}'_{\rm om} - 2\kappa(\Phi'_{\rm om})m(\Phi'_{\rm om})\left(\frac{1}{R_{\rm p}} + \frac{1}{R_{\rm v}}\right) - (\Phi'_{\rm om} - \Phi_{\rm nt})\left[\frac{h_\kappa}{\mathcal{A}'_{\rm om}}\partial_\Phi \kappa(\Phi'_{\rm om}) - \frac{R_{\rm v} h_m}{\mathcal{A}'_{\rm om}}\partial_\Phi(\kappa(\Phi'_{\rm om})m(\Phi'_{\rm om}))\right] , \quad \text{(S24)}$$

where the auxiliary functions h_κ and h_m are defined in Eq. (S21) and we defined

$$\hat{\Sigma}'_{\rm om} = \Sigma + e(\Phi'_{\rm om}) + 2\kappa(\Phi'_{\rm om})m(\Phi'_{\rm om})^2 - (\Phi'_{\rm om} - \Phi_{\rm nt})\partial_\Phi\left(e(\Phi'_{\rm om}) + 2\kappa(\Phi'_{\rm om})m(\Phi'_{\rm om})^2\right) . \quad \text{(S25)}$$

Chemically-generated elastic modulus Within the assumptions of our model, Eq. (S24) is an exact relation that links the pressure difference ΔP to the measured area increase $\Delta\mathcal{A}$. Note that the dependence on $\Delta\mathcal{A}$ enters this expression both through $\Phi'_{\rm om}$ (via Eq. (S13b)) and through the geometric variables $L_{\rm p}$ and $\mathcal{A}'_{\rm om}$ (respectively via Eq. (S18) and via Eq. (S17))[3].

From Fig. 2a it is clear that the aspiration tension depends, in first approximation, linearly on $\Delta\mathcal{A}/\mathcal{A}_{\rm om}$. Given that the measured values of $\Delta\mathcal{A}$ are always below $\sim 25\%$, we can expand Eq. (S24) for small area increases, so to collect information on the coefficient of this linear dependence. For simplicity, we focus now on aspirations that keep the outer membrane segment radius constant, i.e. $R_{\rm v} = R_{\rm om}$. Then, $L_{\rm p}$ is related to $\Delta\mathcal{A}$ via Eq. (S18) as

$$L_{\rm p} = R_{\rm v}\left(\frac{2}{\sin\theta}\frac{\Delta\mathcal{A}}{\mathcal{A}_{\rm om}} - \cos\theta\tan\theta/2\right) . \quad \text{(S26)}$$

By plugging this expression and Eq. (S13b) back into Eq. (S24) and expanding to first order in the area increase, we obtain

$$\frac{\Delta P}{2\left(\frac{1}{R_{\rm p}} - \frac{1}{R_{\rm v}}\right)} \simeq \Sigma_{\rm app} + K_{\rm app}\frac{\Delta\mathcal{A}}{\mathcal{A}_{\rm om}} + O(\Delta\mathcal{A}^2) , \quad \text{(S27)}$$

where $\Sigma_{\rm app}$ is a tension-like term that collects all $\Delta\mathcal{A}$-independent quantities, while $K_{\rm app}$ is the *chemically-generated elastic modulus*, with general structure

$$K_{\rm app} = \Delta\Phi\left(\frac{j_m^{(1)}}{R_{\rm v}}\partial_\Phi(\kappa(\Phi_{\rm om})m(\Phi_{\rm om})) + \frac{j_\kappa^{(1)}}{R_{\rm v}^2}\partial_\Phi\kappa(\Phi_{\rm om})\right) + \Delta\Phi^2\left(\partial_\Phi^2(e(\Phi_{\rm om}) + 2\kappa(\Phi_{\rm om})m(\Phi_{\rm om})^2) + \frac{j_m^{(1)}}{R_{\rm v}}\partial_\Phi^2(\kappa(\Phi_{\rm om})m(\Phi_{\rm om})) + \frac{j_\kappa^{(1)}}{R_{\rm v}^2}\partial_\Phi^2\kappa(\Phi_{\rm om})\right) , \quad \text{(S28)}$$

where we defined the four auxiliary functions

$$j_m^{(1)}(\theta) = 2\left(1 + 2\cos\theta + \sin\theta - 2\cot\theta/2\right) , \quad \text{(S29a)}$$

$$j_\kappa^{(1)}(\theta) = \frac{1}{4\sin\theta}\left(-7 + 7\cos 2\theta + 2\cos 3\theta - 2\cot\theta/2\right) , \quad \text{(S29b)}$$

$$j_m^{(2)}(\theta) = -\left(2 + 2\cos\theta + \sin\theta + \tan\theta/2\right) , \quad \text{(S29c)}$$

$$j_\kappa^{(2)}(\theta) = \frac{7}{4} + \cos\theta + \frac{1}{4(1+\cos\theta)} . \quad \text{(S29d)}$$

which depend only on θ and are displayed in Fig. S5.

As reported in the main text, the measured value for $K_{\rm app}$ from tGPMV aspiration is 3.1 ± 0.2mN/m. Typical parameter values are $m \sim 1/(100{\rm nm})$ for the spontaneous curvature, $\kappa \sim 10^{-19}J$ for the bending rigidity. The typical size of a tGPMV

[3] Note that for constant volume aspirations, where $V'_{\rm om} = V_{\rm om}$ is maintained throughout suction, also $R_{\rm v}$ depends on $\Delta\mathcal{A}$, although this dependence cannot explain the relatively large area increase shown in Fig. 2a, nor the magnitude of $K_{\rm app}$.

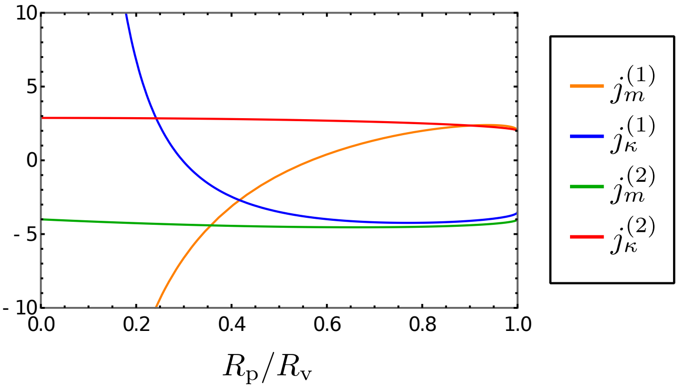

Figure S5: The auxiliary functions $j_\beta^{(\alpha)}$ appearing in Eq. (S28), quantifying the geometric contributions to the chemically-generated elastic modulus $K_{\rm app}$, as a function of $\sin\theta = R_{\rm p}/R_{\rm v}$ for aspirations at constant $R_{\rm v} = R_{\rm om}$.

is $R_{\rm om} \sim 10\mu$m. Roughly, the derivatives of $\kappa(\Phi_{\rm om})$ or $m(\Phi_{\rm om})$ with respect to concentrations quantify *differences* of these material parameters between membrane consisting of pure phases (i.e. bilayers made entirely of either A or B lipids). Hence, it is tempting to estimate their values to be at most of the same order of magnitude as the values for pure membranes. Furthermore, from Fig. S5 we see that the magnitude of the functions appearing in Eq. (S29) is, in modulus, consistently below 10 for $R_{\rm p} \gtrsim R_{\rm v}/5$. Since $|\Delta\Phi| < 1$, we infer that all terms appearing in Eq. (S28) besides the one depending on $e(\Phi_{\rm om})$ will contribute to $K_{\rm app}$ with magnitudes of the order at most of $\sim 10\mu$N/m, i.e. about two orders of magnitude less than the measured value. We therefore conclude that the main contribution to the chemically-generated elastic modulus must come from the internal energy, i.e.:

$$K_{\rm app} \simeq \Delta\Phi^2 \frac{\partial^2 e(\Phi_{\rm om})}{\partial\Phi^2}\,. \tag{S30}$$

It is not possible to find a more precise estimate for $K_{\rm app}$ without choosing an explicit form for the free energy density Eq. (S8), in particular of the exact dependence on Φ of the curvature coupling functions $\kappa(\Phi_{\rm om})$ and $m(\Phi_{\rm om})$.

An explicit model

In our derivation we did not have to provide any detail about the explicit *composition* dependence of the free energy density $f(\Phi, M)$ appearing in Eq. (S8). In fact, this was not necessary in order to prove that, when the tGPMV is aspirated into the pipette, the relative area increase induces a shift in the outer segment composition $\Phi_{\rm om} \to \Phi_{\rm om}{}'$ (see Eq. (S13b)). In turn, this compositional shift generates an elastic term which opposes any further area increase. Such term, quantified in first approximation by the "apparent" elastic modulus $K_{\rm app}$ appearing in Eq. (S28), is the only one that sources a linear response (i.e. a term proportional to $\Delta\mathcal{A}/\mathcal{A}_{\rm om}$) to the applied pressure differential $\Delta P = P_2 - P_1$. We can argue that it is precisely this term that avoids the "droplet-like" instability observed in homogeneous vesicles [2], since now the elastic term can counterbalance arbitrarily high ΔP values, trying to push the outer tGPMV back into its spherical configuration prior to aspiration.

What we cannot do without an explicit expression for $f(\Phi, M)$, however, is to provide an exact quantitative estimate for $K_{\rm app}$ in terms of the membrane material parameters. It is thus instructive to choose a specific form for the free energy density - and specifically for the three functions $e(\Phi)$, $m(\Phi)$ and $\kappa(\Phi)$ -, and show how all the physically relevant quantities, namely the equilibrium composition difference $\Delta\Phi$, the total tension $\hat{\Sigma}_{\rm om}$ and the elastic modulus $K_{\rm app}$ can be computed explicitly.

We need first to fix an explicit form of the curvature-independent free energy density per unit area $e(\Phi)$: a typical choice for qualitative descriptions of binary mixtures is the mean-field theory of two-dimensional lattice gas theory. Then, following e.g. [4], we set

$$e(\Phi) = u(\Phi) - k_B T s(\Phi)\,, \tag{S31}$$

with the internal energy density $u(\Phi)$ and entropy density $s(\Phi)$

$$u(\Phi) = \frac{\omega}{a}\Phi(1-\Phi)\,, \tag{S32a}$$

$$s(\Phi) = -\frac{1}{a}\left(\Phi\ln\Phi + (1-\Phi)\ln(1-\Phi)\right)\,, \tag{S32b}$$

where $\omega > 0$ is the net $A - B$ lipid interaction strength, which promotes demixing, and T is the vesicle temperature. A thermodynamic system described by Eq. (S32) can undergo phase separation for subcritical temperatures $T < T_c = \frac{w}{2k_B}$ and composition values within the binodal interval.

Furthermore, we choose to model the curvature-composition interaction by allowing the spontaneous curvature to be a linear function

$$m(\Phi) = m_B + \Delta m \, \Phi \,, \tag{S33}$$

where $\Delta m = m_A - m_B$, with m_A (m_B) the spontaneous curvature of a membrane consisting purely of A (B) lipids. Conversely, we take for simplicity the bending modulus to be the same for both species, i.e. we set $\kappa(\Phi) = \kappa$. For a general discussion on possible alternatives to Eq. (S33) see [4].

From Eq. (S32) and Eq. (S33) we get the explicit form of the free energy density Eq. (S8):

$$f(\Phi, M) = \frac{\omega}{a}\Phi(1-\Phi) + \frac{k_B T}{a}\left(\Phi \ln \Phi + (1-\Phi)\ln(1-\Phi)\right) + 2\kappa\left(M - m_B - \Delta m \, \Phi\right)^2 \,. \tag{S34}$$

The first derivative of $f(\Phi, M)$ with respect to composition is then

$$\frac{\partial f}{\partial \Phi} = \frac{\omega}{a}(1-2\Phi) - \frac{2k_B T}{a}\tanh^{-1}(1-2\Phi) - 4\kappa\Delta m\left(M - m_B - \Delta m \, \Phi\right) \,. \tag{S35}$$

A curvature coupling of the form Eq. (S33) produces a non-homogeneous vesicle *prior to aspiration*. To see this, we take the derivative with respect to $\Phi_{\rm om}$ and $\Phi_{\rm nt}$ of Eq. (S9), which leads to the chemical equilibrium condition

$$\begin{aligned}
&\frac{\omega}{a}(1-2\Phi_{\rm om}) - \frac{2k_B T}{a}\tanh^{-1}(1-2\Phi_{\rm om}) - 4\kappa\Delta m\left(M_{\rm om} - m_B - \Delta m \, \Phi_{\rm om}\right) \\
&\qquad = \frac{\omega}{a}(1-2\Phi_{\rm nt}) - \frac{2k_B T}{a}\tanh^{-1}(1-2\Phi_{\rm nt}) - 4\kappa\Delta m\left(M_{\rm nt} - m_B - \Delta m \, \Phi_{\rm nt}\right) \,,
\end{aligned} \tag{S36}$$

where $M_{\rm om} = 1/R_{\rm om}$ and $M_{\rm nt} = -1/(2R_{\rm nt})$ are respectively the curvatures of the spherical outer membrane and the cylindrical nanotubes. We can cast this equation in a simpler form by using Eq. (S5), which implies

$$\Phi_{\rm om} = \Phi - (1-y)\Delta\Phi \,, \tag{S37a}$$

$$\Phi_{\rm nt} = \Phi + y\Delta\Phi \,, \tag{S37b}$$

where Φ is the global mole fraction defined in Eq. (S1) and $y = \mathcal{A}_{\rm om}/\mathcal{A}_{\rm tot}$ is the outer segment area fraction. Plugging Eq. (S37) back into Eq. (S36) we get

$$\frac{w}{a}\Delta\Phi + \frac{k_B T}{2a}\ln\left(\frac{\Phi - (1-y)\Delta\Phi}{\Phi + y\Delta\Phi}\,\frac{1 - \Phi - y\Delta\Phi}{1 - \Phi + (1-y)\Delta\Phi}\right) = 2\kappa\Delta m\left(\Delta m \Delta\Phi - M_{\rm nt} + M_{\rm om}\right) \,. \tag{S38}$$

Although it is not possible to find an analytic solution to this equation, we can find an approximate value for weak curvature-composition couplings. In this case we can expanding Eq. (S38) for small Δm and get

$$\Delta\Phi \simeq \kappa\Delta m(M_{\rm nt} - M_{\rm om})\frac{a}{k_B T}\left(\frac{1}{4(1-\Phi)\Phi} - \frac{T_c}{T}\right)^{-1} + O(\Delta m^2) \,, \tag{S39}$$

where $M_{\rm nt}$ and $M_{\rm om}$ are the local mean curvatures of the two domains. As expected, $\Delta\Phi$ vanishes for $\Delta m = 0$. It also vanishes when the geometry of the vesicle is homogeneous ($M_{\rm om} = M_{\rm nt}$) or when the mole fraction is saturated to extremal values ($\Phi = 0, 1$), so that the membrane consists of a single component. In general, Eq. (S39) shows how a curvature-dependent free energy necessarily enforces a difference of equilibrium compositions between different membrane domains - without the need to invoke thermodynamic phase-separation even at subcritical temperatures $T < T_c$ [4].

We can now compute the chemically-generated modulus $K_{\rm app}$ for this specific model. First, note that since $m(\Phi_{\rm om})$ is a linear function and κ is a constant, the general expression Eq. (S28) simplifies considerably:

$$K_{\rm app} = \Delta\Phi\frac{\kappa\Delta m}{R_{\rm v}} j_m^{(1)} + \Delta\Phi^2\left[\frac{k_B T}{a}\left(\frac{1}{\Phi - (1-y)\Delta\Phi} + \frac{1}{1 - \Phi + (1-y)\Delta\Phi}\right) - 4\left(\frac{k_B T_c}{a} - \kappa\Delta m^2\right)\right] \,. \tag{S40}$$

Now, as shown in Fig. S5, $|j_m^{(1)}(\theta)| < 10$ for $R_{\rm p} \gtrsim R_{\rm v}/5$. If we use the approximate values $|\Delta m| \simeq 1/(100{\rm nm})$, $\kappa \simeq 10^{-19} J$ and $R_{\rm v} \simeq 10\mu$m, we get $|\frac{\kappa\Delta m}{R_{\rm v}} j_m^{(1)}| \simeq 1\mu$N/m. Similarly, the $\kappa\Delta m^2$ term will have a magnitude of at most $\simeq 10\mu$N/m.

We thus see that both these terms cannot produce significant contributions to $K_{\rm app}$, whose measured value is much larger, of the order of a few mN/m.

What is left to consider in Eq. (S40) is the contribution originating from the thermodynamic free energy $e(\Phi_{\rm om})$. For small $\Delta\Phi$ this can be approximated as

$$K_{\rm app} \simeq \Delta\Phi^2 \frac{k_B T}{a}\left(\frac{1}{\Phi(1-\Phi)} - 4\frac{T_c}{T}\right) + O(\Delta m^3)\,, \tag{S41}$$

which, at leading order, depends on Δm only through $\Delta\Phi$ (see Eq. (S39)). We can take some putative values for the domain compositions in order to obtain an estimate for $K_{\rm app}$: consider a situation where the nanotubes are almost purely made by A molecules, i.e. $\Phi_{\rm nt} \simeq 1$. From the sorting diagram in Fig. 3b, we can postulate that the outer segment composition is about $\Phi_{\rm om} \simeq 0.65$, so that $\Delta\Phi \simeq 0.35$. Assuming both environments have roughly the same relative area, we can estimate the total mole fraction to lie somewhere in between, say at $\Phi \simeq 0.85$. We then have that Eq. (S41) becomes

$$K_{\rm app} \simeq \left(6.5 - 3.4\frac{T_c}{T}\right) \text{mN/m}\,. \tag{S42}$$

which would require $\omega \simeq 2.1 k_B T$ (or equivalently $T \sim 0.96 T_c$) in order for this expression to reproduce the measured value $K_{\rm app} \simeq 3.1$mN/m, with a lipid cross sectional area of $a \simeq 0.6$nm^2.

References

[1] J.-B. Fournier and P. Galatola. Corrections to the Laplace law for vesicle aspiration in micropipettes and other confined geometries. *Soft Matter*, 4(12):2463, 2008.

[2] Tripta Bhatia, Jaime Agudo-Canalejo, Rumiana Dimova, and Reinhard Lipowsky. Membrane Nanotubes Increase the Robustness of Giant Vesicles. *ACS Nano*, 12(5):4478–4485, may 2018.

[3] Reinhard Lipowsky. Understanding Membranes and Vesicles: A Personal Recollection of the Last Two Decades. In *Physics of Biological Membranes*, pages 3–44. Springer International Publishing, Cham, 2018.

[4] Piermarco Fonda, Melissa Rinaldin, Daniela J. Kraft, and Luca Giomi. Thermodynamic equilibrium of binary mixtures on curved surfaces. *Physical Review E*, 2019.
